# Maximal strength and voluntary activation of adductor pollicis after a single session of acute intermittent hypercapnia or acute intermittent hypoxia

**DOI:** 10.1113/EP093227

**Published:** 2025-09-29

**Authors:** Anandit J. Mathew, Harrison T. Finn, Chiettha Prajnadewie, Simon C. Gandevia, Janet L. Taylor, Jane E. Butler

**Affiliations:** ^1^ Neuroscience Research Australia Sydney New South Wales Australia; ^2^ University of New South Wales Sydney New South Wales Australia; ^3^ Prince of Wales Hospital Sydney New South Wales Australia

**Keywords:** intermittent hypercapnia, intermittent hypoxia, serotonin, transcranial magnetic stimulation, voluntary activation

## Abstract

Acute intermittent hypoxia (AIH) can increase maximal strength of limb muscles in people with incomplete spinal cord injury (SCI), but it is mostly untested in people without SCI. Acute intermittent hypercapnia (AIC) may engage similar respiratory circuits to AIH, but the effects of AIC on human limb motor output are unknown. We examined whether single sessions of AIH or AIC improved motor output to a hand muscle in neurologically intact people. Twelve adults completed a single 30‐min session of AIH (breathing alternate 1‐min low oxygen air and 1‐min normal air), AIC (alternate 1‐min high carbon dioxide air and 1‐min normal air), or SHAM (normal air). At baseline and for 80 min post‐intervention, participants performed repeated isometric maximal voluntary thumb adductions. Transcranial magnetic stimulation elicited motor evoked potentials (MEPs) from first dorsal interosseous and adductor pollicis at each time point. Generalised linear mixed models were compared between conditions (AIH, AIC, SHAM). Normalised to baseline, voluntary activation was higher after AIC than SHAM (5.9%, *P *< 0.001) and AIH (5.5%, *P *< 0.001); MVC force was higher after AIC than SHAM (7.7%, *P *< 0.001), whereas maximal EMG was higher after AIH than SHAM (14.3%, *P *< 0.001). MEPs and maximal M‐waves did not differ between conditions for either muscle (*P *> 0.25). Thus, single sessions of AIC induced small motor output improvements in people without SCI, but AIH did not. AIC increased maximal voluntary activation, but the mechanisms for this remain unclear because the MEPs provided no evidence for corticospinal facilitation.

## Abstract (highlights)

- **What is the central question of this study?**
  Do single sessions of acute intermittent hypoxia (AIH) or acute intermittent hypercapnia (AIC) facilitate hand muscle voluntary activation and force in neurologically intact adult humans?
- **What is the main finding and its importance** **?**
  AIC improved maximal voluntary activation and force by 5–6%, while AIH did not.

## 1 INTRODUCTION

Acute intermittent hypoxia (AIH) is an experimental treatment that may improve limb muscle strength and function in people with chronic incomplete spinal cord injury (SCI) after repeated sessions (Hayes et al., 2014; Sandhu et al., 2019; Tan et al., 2021; Trumbower et al., 2012) or even after a single 30‐min session of exposure (Sandhu et al., 2019; Trumbower et al., 2012). However, when testing the excitability of the motor pathway in people without spinal cord injury, the results after a single AIH session are mixed. One study indicates increased corticospinal excitability (Christiansen et al., 2018), but these results have not been replicated by others (Finn et al., 2022; Mathew et al., 2024; Radia et al., 2022; Welch et al., 2022; for review see Gandevia & Butler, 2024).

A single session of AIH typically consists of breathing 60–120 s periods of hypoxic air (8–10% fraction of inspired oxygen; $F_{iO_{2}}$) alternating with similar periods of breathing normoxic air (21% $F_{iO_{2}}$) over 30 min. AIH intermittently stimulates the carotid bodies, which subsequently activate neurons which project to the spinal cord from the medullary raphe nuclei to release serotonin onto the motoneurones (e.g., Hoffman & Mitchell, 2011; MacFarlane et al., 2008; Perim et al., 2019). Decades of research using AIH in animals have focused on AIH‐induced facilitation of phrenic nerve output and suggest that AIH induces motor neuroplasticity via intermittent release of serotonin and adenosine (Hoffman & Mitchell, 2011; MacFarlane et al., 2008; Perim et al., 2019). Serotonin and adenosine release is followed by new protein synthesis on the post‐synaptic motoneurone membrane and enhanced motoneuronal synaptic function (Baker‐Herman & Mitchell, 2002; Dale et al., 2017), but at higher levels of hypoxia the serotonin‐driven phrenic neuroplasticity is inhibited by the competing mechanism of neuroplasticity induced by adenosine (Perim et al., 2017, 2018). However, mechanisms in humans for AIH‐induced motor facilitation are not yet clear. In addition, there are mixed results from human studies that attempt to induce facilitation of respiratory and limb motor output in both neurologically intact and spinal cord‐injured populations.

AIH‐induced motor facilitation has been thought to involve the postsynaptic membrane of the motoneurone (Christiansen et al., 2018; Dale‐Nagle et al., 2010a, 2010b). Two human studies have demonstrated corticospinal facilitation for limb muscles after AIH (tested with transcranial magnetic stimulation [TMS] induced motor evoked potentials [MEP]) (Christiansen et al., 2021, 2018) in people without and with SCI, while one study has demonstrated increased motor unit firing rates after AIH (Pearcey et al., 2024) in people with chronic incomplete SCI. However, clear facilitation of limb muscle MEPs has not been replicated in a neurologically intact population (Finn et al., 2022; Mathew et al., 2024; Radia et al., 2022), nor were diaphragm MEPs facilitated (Welch et al., 2022, 2021). Furthermore, AIH has marginal or no effects on spinal reflexes, suggesting that neither the Ia afferent pathway nor the motoneurones are consistently facilitated (Bogard et al., 2024; Finn et al., 2022; Tan et al., 2024). In people with chronic incomplete SCI, AIH‐induced limb motor facilitation has typically been demonstrated using certain measures of motor output like strength and motor performance in both the upper (Afsharipour et al., 2023; Sandhu et al., 2021) and lower limbs (Hayes et al., 2014; Lynch et al., 2017; Sandhu et al., 2019; Tan et al., 2021; Trumbower et al., 2012). The effects of AIH in people without SCI have been investigated in two recent studies, but the results remain uncertain (Bogard et al., 2024; Smith & Salmon, 2025). In one, short‐lived (15 min) improvements in hand grip force and associated EMG (by 10–15%) are reported after a single session of AIH (Smith & Salmon, 2025), but voluntary activation was not assessed. In the other, after four daily sessions of AIH, minor improvements are reported for the central activation ratio (an alternative index of voluntary activation) measured during maximal elbow flexor contractions prior to a fatigue protocol (pre‐AIH 98.71 ± 2.17%, post‐AIH 99.13 ± 1.83; i.e. a difference of ~0.4%) (Bogard et al., 2024). However, there was no sham intervention, and maximal torque and EMG did not increase (Bogard et al., 2024).

The addition of carbon dioxide to AIH may help to improve the consistency of outcomes as it stimulates a greater ventilatory motor response (Mateika et al., 2024; Panza et al., 2023; Welch et al., 2022). Controlling baseline levels of CO_2_ before and during AIH interventions has increased the augmentation of ventilation (Panza et al., 2022; Vermeulen et al., 2020). Acute intermittent hypercapnic hypoxia (AIHH; i.e., period of hypercapnic hypoxia alternated with a period of normocapnic normoxia) facilitated diaphragmatic MEPs, which was not seen after AIH alone (Welch et al., 2022). However, AIHH does not have a lasting effect on ventilation in either awake (Diep et al., 2007) or asleep humans (Deacon et al., 2017).

Similar to hypoxia, hypercapnia activates the serotonergic raphe nuclei, the cells of which project to the spinal cord (Corcoran et al., 2013; Mitchell et al., 2008; Veasey et al., 1995, 1997) and could potentially induce serotonin‐dependent plasticity without concurrent hypoxia. Increased density of serotonergic axons in denervated phrenic circuitry has been observed in rodents following daily intermittent normoxic hypercapnia training ($F_{\mathrm{iC}O_{2}}$ = 7%) for 2 weeks, suggesting that the intermittent normoxic hypercapnia could promote phrenic plasticity (Randelman, 2021).

Repeated localised intracarotid hypercapnia activating the carotid chemoreceptors resulted in long‐term facilitation of phrenic nerve output in cats (Morris et al., 1996), but systemic hypercapnia has different effects. In rodents, severe sustained systemic hypercapnia does not facilitate or depress respiration (Stipica et al., 2016), whereas intermittent normoxic hypercapnia ($F_{\mathrm{iC}O_{2}}$ = 10–15%) alone (Bach & Mitchell, 1998; Stipica Safic et al., 2018; Valic et al., 2016) or sustained hyperoxic hypercapnia ($F_{\mathrm{iC}O_{2}}$ ∼2–3 mmHg above CO_2_ apnoeic threshold) (Baker et al., 2001) can depress phrenic output. Humans showed no respiratory facilitation or depression in the 30 min after 40 min of intermittent hypercapnia (40 s hypercapnia and 20 s of normocapnia) (Shing et al., 2023). However, the effect of intermittent normoxic hypercapnia (acute intermittent hypercapnia; AIC) on limb motor output has not been explored in humans or animals. Nevertheless, human limb motoneurones show increased sustained firing after peripheral nerve stimulation during hypercapnia (Hatano et al., 2022). This change is consistent with serotonergic actions on the motoneurones, and so opens the possibility that intermittent hypercapnia could result in delivery of intermittent serotonin to limb motoneurones and hence induce serotonin‐dependent plasticity in the spinal cord.

Thus, the aims of this study in neurologically intact people were (1) to determine whether AIH improves measures of limb motor output (MVC, voluntary activation and TMS MEPs) and (2) to determine the effects of AIC on limb motor output (MVC, voluntary activation and TMS MEPs). We hypothesised that both AIH and AIC would improve limb motor output.

## 2 METHODS

### 2.1 Participants

This study was approved by the University of New South Wales Human Research Ethics Committee (HC200664) and conducted in accordance with the *Declaration of Helsinki* (2013), except for database registration. Thirteen healthy neurologically intact participants (five male) were recruited by local advertisement, and informed written consent was obtained. Participants did not undergo any screening. Sample size and power calculations were conducted based on the effect sizes for strength increases after AIH in people with SCI from Trumbower et al. (2012; *d*_z_ = 2.1) and Lynch et al. (2017; *d*_z_ = 1.3), and also increases in TMS‐evoked MEPs in neurologically intact participants from Christiansen et al. (2018; *d*_z_ = 0.95). An effect size of 0.95 required 11 participants for a power of 0.8 and α of 0.05 for within subject comparisons. The participants had a mean age (±SD) of 32.9 ± 9.5 years (range: 23–53), and a mean body‐mass index of 21.9 ± 3.0 kg/m^2^ (range: 16.9–27.2). Exclusion criteria included pregnancy, any neurological, cardiac or respiratory disease, use of selective serotonin reuptake inhibitors, age >55 years, or a family history of epilepsy. One male participant's data were excluded from all three experiment days due to an inability to consistently perform the experimental contractions according to instructions.

### 2.2 Experimental design

A randomised crossover study design was used to measure the effects of poikilocapnic acute intermittent hypoxia (AIH), acute intermittent normoxic hypercapnia (AIC) and a sham intervention (breathing normoxic room air; SHAM) on voluntary drive (strength, voluntary activation and EMG) of the adductor pollicis muscle (AP; thumb adduction). Corticospinal excitability was also assessed using MEPs evoked by TMS from the first dorsal interosseus (FDI) and AP muscles. Each participant visited the laboratory three times, once for each of the three conditions in which the fractions of inspired oxygen ($F_{iO_{2}}$) and carbon dioxide ($F_{\mathrm{iC}O_{2}}$) were controlled: (1) AIH: $F_{iO_{2}}$ = 9%, $F_{\mathrm{iC}O_{2}}$ ∼0.05% alternating with room air $F_{iO_{2}}$ = 21%, $F_{\mathrm{iC}O_{2}}$ ∼0.05%; (2) AIC: $F_{iO_{2}}$ = 21%, $F_{\mathrm{iC}O_{2}}$ = 7%, alternating with room air $F_{iO_{2}}$ = 21%, $F_{\mathrm{iC}O_{2}}$ ∼0.05%; and (3) SHAM: room air $F_{iO_{2}}$ = 21%, $F_{\mathrm{iC}O_{2}}$ ∼0.05%. Since AIH is known to produce effects lasting up to several hours, there was at least a week between visits (Christiansen et al., 2018; Trumbower et al., 2012). The order of the interventions was randomised at the first visit. Only the investigator who controlled the switching of the gases during the intervention was unblinded to the intervention gases. The two other investigators (one to hold the coil for TMS and one to provide encouragement during contractions) were blinded to the intervention. Participants were instructed to complete the same routine of sleep, exercise, food and caffeinated beverages in the 12 h prior to each experimental session. All experiments for an individual were conducted at the same time of day.

### 2.3 Experimental set‐up

**FIGURE 1:**
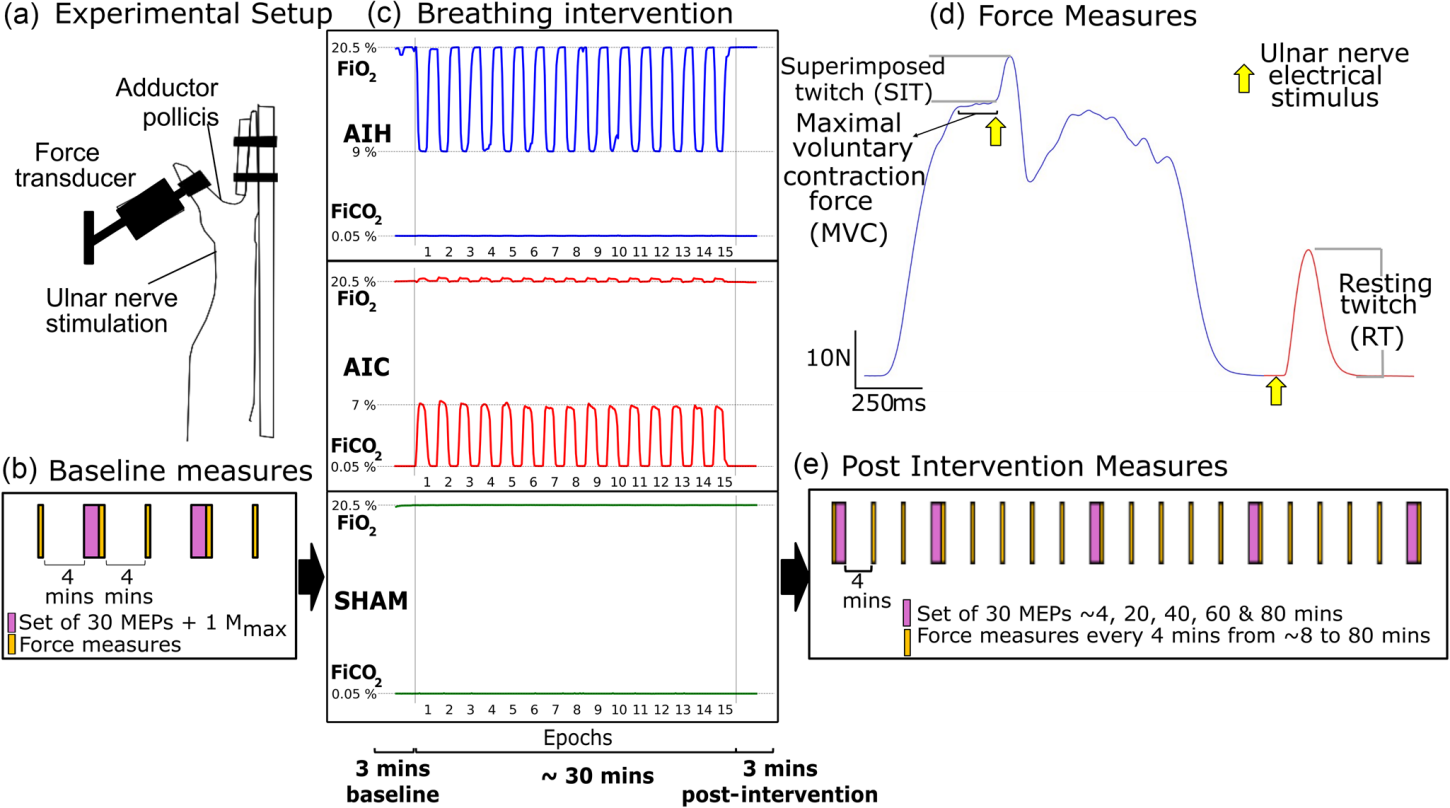
(a) Experimental set‐up for adductor pollicis force measures, *n* = 12 (4 males). (b) Experimental protocol for baseline measures, which include maximal force and voluntary activation, and sets of 30 motor evoked potentials (MEPs). (c) One of three breathing interventions was delivered in each experimental session. Breathing interventions: acute intermittent hypoxia (AIH), acute intermittent hypercapnia (AIC) and normoxic room air (SHAM). Traces show the fraction of inspired oxygen ($F_{iO_{2}}$) and fraction of inspired carbon dioxide ($F_{\mathrm{iC}O_{2}}$) delivered to the participants during the 30‐min AIH, AIC and SHAM breathing periods. Note that in each of the 15 epochs, the low oxygen or high carbon dioxide gas mixture was delivered for 1 min followed by 1 min of air. (d) Sample trace of force measures depicting the measured parameters: peak force during a maximal voluntary contraction (MVC), superimposed twitch (SIT) and resting twitch (RT) produced by supramaximal nerve stimulation. (e) Experimental protocol for post‐intervention measures (see Methods).

The participant was seated in a chair with the forearm of their dominant arm (left dominant, *n* = 1) resting on a table, approximately at heart level, with the forearm mid‐prone, upper arm at ∼45°, and shoulder relaxed in a neutral position. The fingers were secured with a padded plate in full extension, and the wrist and forearm were secured with Velcro straps to a rigid support (Figure 1a). This set‐up reduced the contribution of other hand muscles during maximal contractions of the adductor pollicis (e.g., D'Amico et al., 2018). The thumb was abducted and placed into a padded ring that was rigidly coupled with a calibrated linear strain gauge (Xtran‐S1W 350N loadcell, Applied Measurement, Melbourne, Australia). Visual feedback of the force was provided to the participant on a monitor.

Force signals were sampled at 4 kHz, while all other signals were sampled at 2 kHz via a 16‐bit analogue‐to‐digital data acquisition interface (Power1401‐3A, Cambridge Electronic Design, Cambridge, UK) and recorded with Spike2 software (v7.12, Cambridge Electronic Design). The participants were instructed to stay awake, to relax between maximal efforts and to avoid moving the target muscles as much as possible during the experiment. During sets of TMS, the investigators and the participant remained silent to minimise changes in arousal.

### 2.4 Electromyographic recordings

Electromyographic (EMG) recording electrodes (Ag–AgCl, 20 mm diameter, Cleartrace, CONMED, Utica, NY, USA) were placed over the FDI and the AP muscles on the dominant hand. For the FDI, electrodes were placed over the belly and over the second metacarpophalangeal joint, with an interelectrode distance of ∼20 mm. The AP electrodes were placed over the muscle belly and the first metacarpophalangeal joint, with an interelectrode distance of ∼20 mm. Electromyographic (EMG) signals were amplified (×1000) and band‐pass filtered (10 Hz to 1 kHz; 1902 isolated pre‐amplifier, Cambridge Electronic Design).

### 2.5 Transcranial magnetic stimulation

A round coil (High Power 90 mm Coil 9784‐00, Magstim 2002, Magstim Company, Whitland, UK) was used to elicit MEPs in the dominant FDI and AP muscles. The round coil was held to the scalp over the vertex (side ‘A’ facing upwards except for the left‐handed participant) to induce a posterior‐to‐anterior current in the contralateral motor cortex. The position was initially localised to elicit the largest MEP in FDI at rest at 30–60% of the magnetic stimulator output. Coil positions were marked on the scalp or tight‐fitting cap worn by the participant to ensure consistent coil placement throughout the experiment. The stimulation intensity for TMS was set at a level that evoked MEPs in the FDI of average amplitude of ∼1 mV in the baseline sets for comparison with previous studies (∼8% *M*_max_ and corresponding to 62 ± 11% stimulator output). MEPs were also recorded at the same time from AP for comparison with the strength and voluntary activation of this muscle, and their size was ∼0.6 mV (∼18% *M*_max_). The stimulus intensity was constant for the experimental session. Each set of MEPs comprised 30 stimuli delivered 5 s apart.

### 2.6 Peripheral nerve stimulation

Electrical stimulation of the ulnar nerve at the wrist was used to evoke maximal M‐waves (*M*_max_) in the AP and FDI muscles and to evoke twitches of the AP. Surface electrodes (Ag–AgCl, 20 mm diameter, Cleartrace, CONMED) were placed with the cathode ∼20 mm proximal to the wrist and lateral to the tendon of flexor carpi ulnaris and the anode ∼30 mm proximal to the cathode. The nerve was stimulated with a constant current stimulator (pulse width 0.2 ms; DS7AH stimulator; Digitimer, Welwyn Garden City, UK). Stimulation intensity was increased in 5 mA increments until the amplitude of the M‐wave for AP plateaued with no increase in amplitude with further increases in current (*M*_max_). The stimulator was then set at 130% of the stimulus intensity that was required to produce *M*_max_ in the AP (55 ± 15 mA). To evoke the superimposed twitch and the resting twitch, pairs of stimuli with an inter‐pulse interval of 10 ms were used.

A single stimulus at this intensity was used to elicit an *M*_max_ in FDI and AP after each set of MEPs. The amplitude and area of *M*_max_ were used to normalise the MEPs of the corresponding set, to account for any peripheral changes that may have resulted from the intervention (Lefebvre et al., 2004).

### 2.7 Voluntary activation and twitch parameters

The participant was instructed to perform brief (∼4 s) isometric maximal voluntary contractions (MVCs) of the AP (thumb adduction). Verbal encouragement and visual feedback of the force signal were given during each contraction. During each MVC, a pair of supramaximal stimuli (see section 2.6, ‘Peripheral nerve stimulation’) was used to elicit a superimposed twitch (SIT). Once the participant relaxed after the contraction (∼5 s), a resting twitch (RT) was elicited using the same stimulation. Voluntary activation was calculated using the ratio of the SIT and subsequent RT (e.g., Allen et al., 1995; Bellemare & Bigland‐Ritchie, 1984; Merton, 1954).

### 2.8 Experimental protocol

At baseline, participants performed five MVCs of the thumb adductors, each with a SIT and RT delivered to assess voluntary activation. MVCs were performed 4 min apart to avoid fatigue with the repeated contractions (e.g., Allen et al., 1997, 1994; for review see: Carroll et al., 2017). A set of MEPs (*n* = 30) and a *M*_max_ were recorded after 4 min of rest following the first MVC, and a second baseline set of MEPs and *M*_max_ was performed 4 min after the third MVC (Figure 1b). Following the baseline measurements, the breathing protocol was administered (see section 2.10, ‘Breathing protocols’).

For the post‐condition measures, an attempt was made to start the MVC measures by ∼4 min after the breathing protocol. However, it was possible only in 12 out of 36 sessions because of the time taken to remove the mask after 3 min of breathing recorded post‐intervention and prepare for the MVC; therefore, for the analysis, the 4‐min measure was excluded, and the 8‐min measure was used as the first post‐intervention measure. MVCs with SITs and RTs were recorded every 4 min from 4–8 min to 80 min after the breathing protocol ended. A set of 30 MEPs followed by a *M*_max_ was recorded at ∼8, 20, 40, 60 and 80 min post‐intervention (Figure 1e).

### 2.9 Breathing set‐up

Participants were fitted with an appropriately sized oro‐nasal facemask (Resmed‐ Quattro‐FX non‐vented) attached to a bacterio‐viral filter and a two‐way valve (Series 2600; Hans Rudolph, Shawnee, KS, USA) that isolated the inspiratory and expiratory circuits.

The inspiratory port of the two‐way valve was connected to bags filled with 240 L of one of the gas mixtures via a three‐way stopcock (series 2100; Hans Rudolph). The gas mixtures were (i) AIH – normocapnic hypoxic air ($F_{iO_{2}}$ = 9%, $F_{\mathrm{iC}O_{2}}$ ∼0.05%); (ii) AIC – hypercapnic normoxic intervention gas ($F_{\mathrm{iC}O_{2}}$ = 7%, $F_{iO_{2}}$ = 21%); or (iii) SHAM – normocapnic normoxic room air ($F_{\mathrm{iC}O_{2}}$ ∼0.05%, $F_{iO_{2}}$ = 21%), while other bags were filled with normoxic room air ($F_{\mathrm{iC}O_{2}}$ ∼0.05%, $F_{iO_{2}}$ = 21%). The AIH bags were filled using a pressure swing hypoxia generator (Hypoxico Inc., hyp‐123, New York) while the AIC bags were filled with a mix of room air ($F_{iO_{2}}$ = 21%), 100% CO_2_ and 100% O_2_ titrated to achieve the required concentrations.

A pneumotachometer (Series 3813; Hans Rudolph), was fitted on the inspiratory side of the two‐way valve. It measured inspiratory flow continuously (DP15‐34; Validyne Engineering, Northridge, CA, USA).

### 2.10 Breathing protocols

After a few minutes to acclimatise to the mask, each participant was given 3 min of normocapnic normoxic room air (CO_2_ ∼0.05%, $F_{iO_{2}}$ = 21%) to record baseline cardiorespiratory measures during quiet breathing ($S_{pO_{2}}$, heart rate, flow and tidal volume). The AIH and AIC protocols were 30 min long and comprised 15 epochs with a minute of the condition gas (hypoxic or hypercapnic) followed by a minute of room air. The unblinded investigator switched between the bags filled with conditioned gas and room air with a manual stopcock. A safety threshold of below 75% $S_{pO_{2}}$ was set to switch the participant back to room air before the end of the 1‐min gas exposure. This threshold was not reached in any participant. The SHAM intervention was 30 min of normocapnic normoxic room air switched every minute between two bags to mimic the AIH and AIC interventions. After the 15 epochs, an additional 3 min of post‐intervention cardiorespiratory signals were recorded with the participant breathing room air (CO_2_ ∼0.05%, $F_{iO_{2}}$ = 21%).

### 2.11 Cardiorespiratory measurements

Cardiorespiratory signals were recorded during the breathing intervention period to record the response to the breathing condition as well as to ensure safety. The inspiratory flow signal was integrated (DP45‐16; Validyne Engineering, Northridge, USA) to give inspired tidal volume (*V*_t_). Respiratory rate (RR) and minute ventilation (${\dot{V}}_{E}$) were calculated off‐line from the volume signal. Fractional end‐tidal CO_2_ ($ET_{CO_{2}}$; Normocap; Datex Instrumentarium, Helsinki, Finland) and fraction of inspired CO_2_ ($F_{\mathrm{iC}O_{2}}$; Normocap; Datex Instrumentarium) as well as inspiratory oxygen concentration ($F_{iO_{2}}$) and expiratory oxygen concentration ($F_{eO_{2}}$) (FDO2, PyroScience GmbH, Germany) were recorded continuously during the intervention.

Pulse oximetry (Nellcor N‐600x Pulse Oximeter with OxiMax, Skaneateles Falls, NY, USA) was used continuously to record the mean peripheral oxygen saturation ($S_{pO_{2}}$) of the non‐dominant index finger. $S_{pO_{2}}$ was averaged by the device over 30–40 s which resulted in a delay of the $S_{pO_{2}}$ signal and potential underestimation of nadir $S_{pO_{2}}$. Mean heart rate was the recorded average over every five beats.

### 2.12 Data analysis

Data processing and analysis were done with Python (v 3.11.4). The cardiorespiratory and electrophysiology data were separated and analysed independently. All data files and sessions were deidentified to blind the analysis. The main outcomes after each condition are voluntary activation and maximal voluntary contraction force, and the supporting outcome measures are EMG_rms_ during the MVC, motor evoked potentials elicited by transcranial magnetic stimulation and resting twitch. We also monitor and report cardiorespiratory responses during the interventions.

#### 2.12.1 Force measures

We aimed to perform the MVC and voluntary activation trials every 4 min from 4–8 to 80 min post‐condition; however, the protocol was prone to cumulative delays and delayed time points. To ensure the discrete time of each measure, the data for the MVC trials were allocated to a time point based on their actual time from the end of the breathing intervention (AIH/AIC/SHAM) using a 2‐min period on either side of the intended time point. If two trials occurred in the same time point window, then their measures were averaged to represent that time point (50 out of the 720 time points).

MVCs of thumb adduction were measured as the maximal force in a window of 1000 ms before the SIT stimulus. The amplitudes of the SIT and RT (peak force of twitch relative to force just prior to the twitch onset) were measured, accounting for electromechanical delay (∼14–19 ms, measured for each person). Voluntary activation was calculated using the following equation $\mathrm{VA}=(1-\frac{\mathrm{SIT}}{\mathrm{RT}})\times 100$ (e.g., Allen et al., 1995; Bellemare & Bigland‐Ritchie, 1984; Merton, 1954). The baseline and post‐intervention MVC, voluntary activation and RT were normalised to the largest of their five baseline values.

#### 2.12.2 EMG measures

The root mean square (RMS) of the adductor pollicis EMG (AP EMG_rms_) was measured for a 100‐ms window before the time of maximal force. The values were normalised to the largest of their five baseline values. Amplitudes and areas for every MEP from FDI and AP were measured and normalised to the amplitude and area of the corresponding *M*_max_. The average MEP amplitude and area normalised to *M*_max_ were then calculated for each set of MEPs. MEPs were removed if there was background EMG activity before the TMS stimulus (52 out of 7560 stimuli). Due to the proximity of the AP EMG electrodes to one another, there was shorting of the AP EMG electrodes during the MVC task on 53 of 900 trials, affecting data from six participants. The EMG from these trials were removed from the analysis.

#### 2.12.3 Breathing measures

**FIGURE 2:**
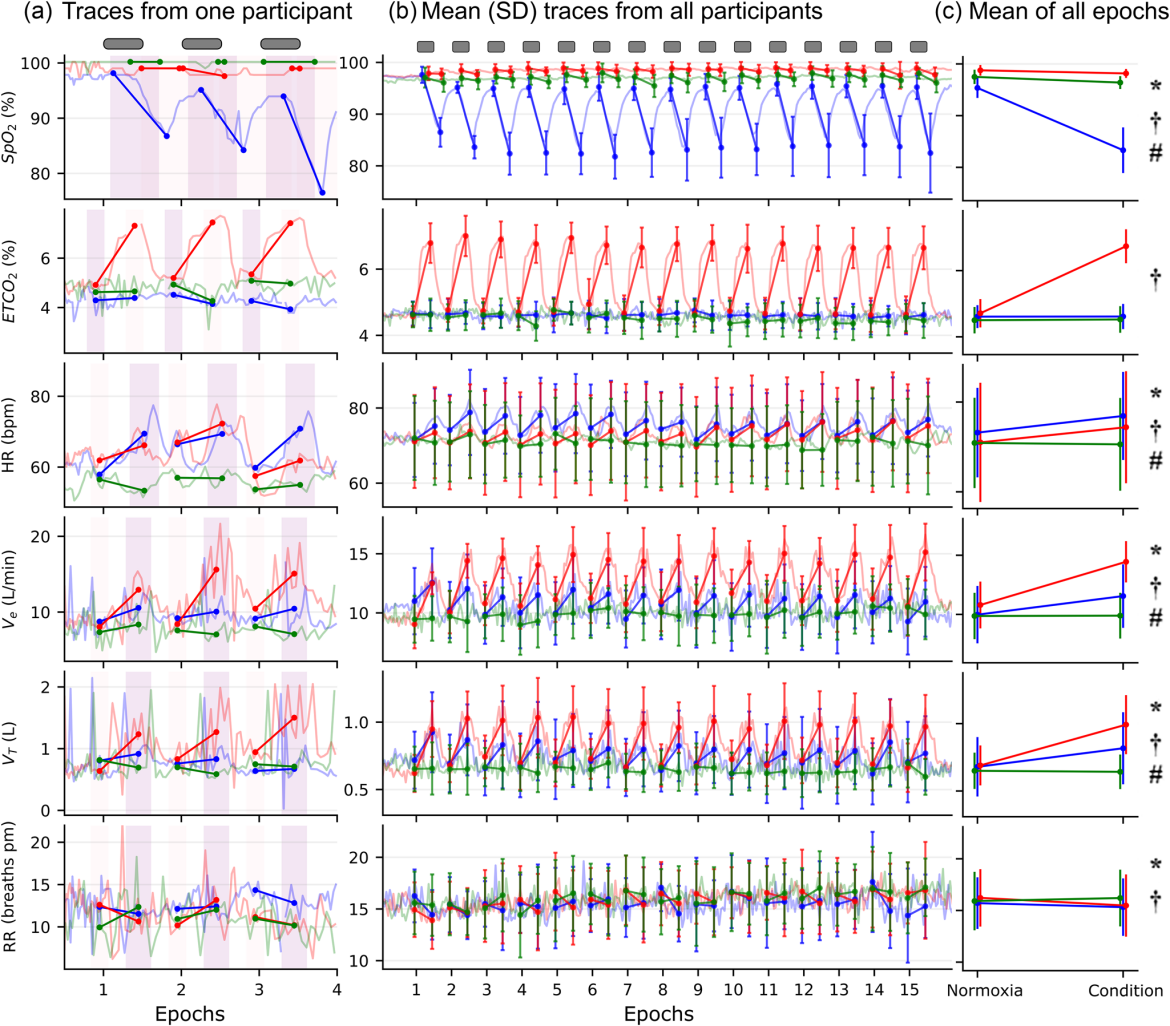
Cardiorespiratory responses during the three breathing interventions – acute intermittent hypoxia (AIH, in blue), acute intermittent hypercapnia (AIC, in red) and sham (SHAM, in green). Grey bars at the top of (a, b) indicate the minute of condition gas. (a) Sample traces for one person, for the first 3 epochs. Windows where measurements were made for each parameter are indicated with pink shading. Note the delay on the $S_{pO_{2}}$ trace due to the instrument delay (see ‘Methods’). (b) Mean (SD) with the mean trace from all participants (*n* = 12, 4 male) for the 30 min of breathing AIH, SHAM and AIC. (c) Mean (SD) across all epochs during normoxia and breathing the condition gas mixture. Independent generalised linear mixed models (GLMM) were used to compare the responses for each parameter during AIH, AIC and SHAM conditions. *P < 0.05 for Bonferroni corrected significant *post hoc* tests for AIH versus SHAM; †*P* < 0.05 for AIC vs SHAM; #*P* < 0.05 for AIH versus AIC. $ET_{CO_{2}}$, end tidal carbon dioxide; HR, heart rate; RR, respiratory rate; $S_{pO_{2}}$, oxygen saturation; ${\dot{V}}_{E}$, minute ventilation, *V*_T_, tidal volume.

The breath‐by‐breath data for volume were used to calculate ventilation as a product of tidal volume and respiratory rate. Mean HR and $S_{pO_{2}}$ as well as peak $ET_{CO_{2}}$ were calculated for each breath. To quantify the change in respiratory measures during AIH or AIC, the intervention period (30 min) was divided into 15 ∼2‐min epochs each comprising 1 min of hypoxic or hypercapnic breathing and 1 min of normoxic breathing. For the SHAM condition, the 30‐min intervention period was divided into ∼2‐min epochs (15 epochs). Each respiratory measure was plotted against the percentage time of each epoch to allow comparison between epochs of slightly different lengths. During each of these 2‐min epochs, the average hypoxic/hypercapnic and normoxic data were measured in time windows set to capture the changes in cardiorespiratory parameters. The time windows are indicated for each signal in Figure 2a and were used for each condition, including SHAM.

### 2.13 Statistical analysis

The statistical analyses were done using SPSS 24 software (v24.0.0.1, IBM Corp., Armonk, NY, USA). Baseline measures for MVC, voluntary activation, resting twitch, EMG_rms_ and MEPs (amplitude and area) were tested for normality using the Shapiro–Wilk test. Baseline values for each parameter were compared across the three conditions (AIH, SHAM, AIC) using repeated‐measures one‐way ANOVAs for normally distributed data (raw or log‐transformed) and Friedman's test for data that were not normally distributed. Generalised linear mixed models were used to compare the effects of the three experimental conditions, that is, AIH, AIC and SHAM. The fixed effects were the condition (AIH, SHAM and AIC), time point (baseline and every 4 min from 8 to 80 min post‐intervention for force measures, and baseline, 8, 20, 40, 60, 80 min post‐intervention for *M*_max_ and TMS evoked MEPS in FDI and AP) and their interaction on the MVC, VA, RT, EMG_rms_ for AP, and MEP and *M*_max_ for FDI and AP. Separate generalised linear mixed models (similar to above) were run on the force data after removing outliers identified using *Z*‐score for data that were normally distributed and modified *Z*‐score method for data that were not normally distributed.

Generalised linear mixed models were also used to compare the mean differences in each epoch for the cardiorespiratory parameters ($S_{pO_{2}}$, heart rate, ventilation, tidal volume, respiratory rate and $ET_{CO_{2}}$) during the conditions. The fixed effects were condition (AIH, AIC, SHAM), epoch (1–15), with interaction effects of condition and epoch. For each parameter, Student's paired *t*‐test was used to compare the baseline and post‐intervention 3‐min breathing periods for AIH and AIC.

**FIGURE 3:**
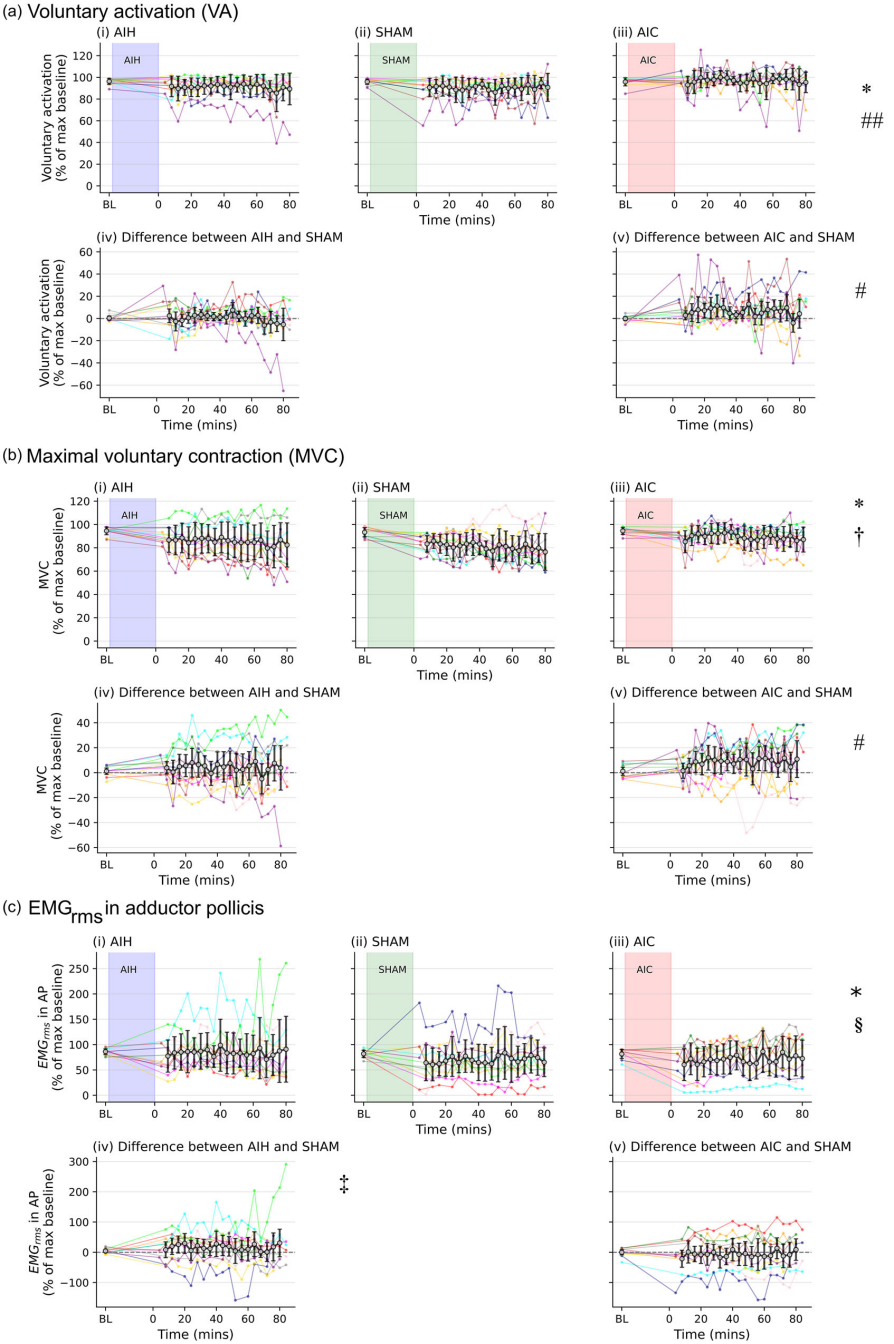
Voluntary activation (a), force (b) and EMG (c) during maximal voluntary contractions of adductor pollicis pre‐ and post‐intervention. Data for individual participants (*n* = 12, 4 males, coloured lines) and mean (SD, black lines) are shown in subpanels (i), (ii) and (iii). Data for baseline (BL) and every 4 min from 8 min to 80 min after acute intermittent hypoxia (AIH) (i), SHAM (ii) or acute intermittent hypercapnia (AIC) (iii) breathing protocols. Subpanels (iv) and (v) show differences between AIH and SHAM (AIH minus SHAM; iv) and AIC and SHAM (AIC minus SHAM; v). In subpanels (iv) and (v), data for individual participants (*n* = 12, 4 males, coloured lines) and mean (±95% confidence interval [CI], black lines) are shown. (a) Voluntary activation is expressed normalised to the largest baseline value. (b, c) Maximal voluntary forces (MVC) (b) and maximal root mean square EMG (EMG_rms_) (c) values are normalised to the largest baseline value. Generalised linear mixed models were used to compare the effects of AIH, SHAM and AIC on MVC, voluntary activation and EMG_rms_. A *P*‐value < 0.05 was taken as statistically significant. *An effect of condition; †an effect of time; #AIC greater than SHAM; ##AIC greater than AIH; ‡AIH greater than SHAM; §AIH greater than AIC.

**FIGURE 4:**
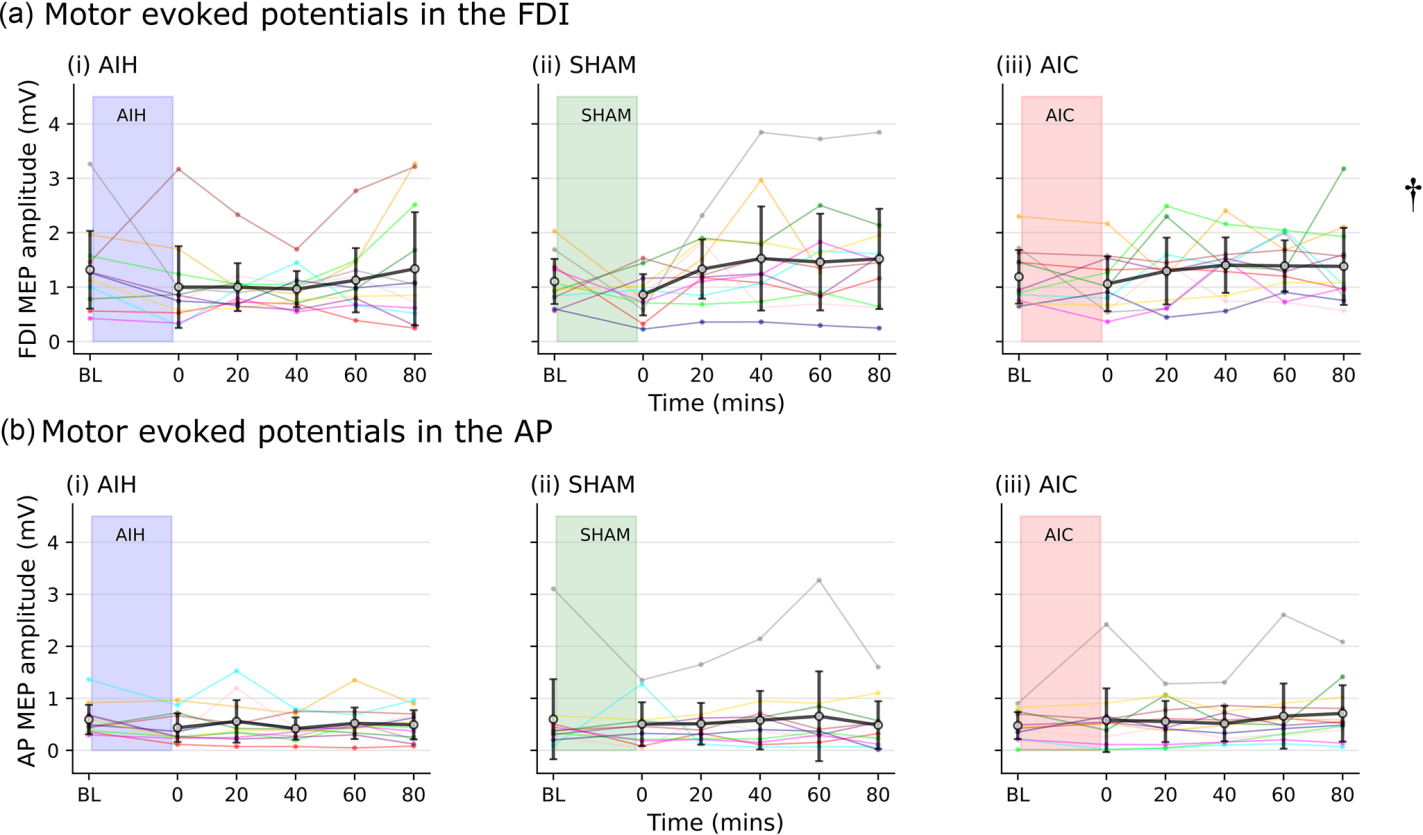
Non‐normalised motor evoked potential (MEP) amplitude for the first dorsal interosseous (FDI; a) and adductor pollicis (AP; b). Data from individual participants (*n* = 12, 4 males, coloured lines) and the group mean (±SD, black lines) for acute intermittent hypoxia (AIH; subpanel (i)), SHAM (subpanel (ii)) and acute intermittent hypercapnia (AIC; subpanel (iii)). There were no significant differences in FDI or AP MEP amplitude at any time point between AIH, SHAM and AIC. A *P*‐value < 0.05 was taken as significant. †An effect of time.

All data are presented in the text and figures as means (SD), and *P* < 0.05 was regarded as statistically significant. *Post hoc* comparisons are expressed as estimated marginal means [confidence interval] (EMM [CI]) with their Bonferroni corrected *P*‐values (*P* < 0.05 taken as significant). In some cases, where indicated, 95% CI are shown. Participant data are shown with consistent colour coding for each participant in Figures 3 and 4.

## 3 RESULTS

### 3.1 Baseline measures

At baseline, there were no differences in maximal voluntary force, voluntary activation, resting twitches, maximal adductor pollicis EMG or MEP and *M*_max_ measures from FDI and AP between the three conditions (AIH, SHAM, AIC) (see Table 1).

**TABLE 1:** Baseline values for force (maximal voluntary contraction force), voluntary activation, resting twitch, maximal adductor pollicis EMG (AP EMG_rms_), and MEP and *M*_max_ amplitude and area on the acute intermittent hypoxia (AIH), sham (SHAM) and acute intermittent hypercapnia (AIC) days.

Baseline values (mean ± SD)		AIH	SHAM	AIC	Test	ANOVA/Friedman
**Maximum voluntary contraction force (N)**		72.9 ± 19.9	72.6 ± 20.0	70.2 ± 19.2	ANOVA	*F* (2, 22) = 0.300, *P* = 0.744
**Voluntary activation (%)**		91.8 ± 6.2	89.8 ± 7.5	86.5 ± 11.5	Friedman's test	χ^2^ = 5.167, *P* = 0.076
**Resting twitch (N)**		17.3 ± 4.8	17.2 ± 5.4	17.5 ± 4.0	ANOVA (log)	*F* (2, 22) = 0.154, *P* = 0.859
**AP EMG_rms_ (mV)**		0.30 ± 0.14	0.31 ± 0.17	0.33 ± 0.25	Friedman's test	χ^2^ = 0.200, *P* = 0.905
**FDI**	MEP amplitude (mV)	1.31 ± 0.74	1.13 ± 0.42	1.15 ± 0.49	ANOVA (log)	*F* (2, 22) = 0.804, *P* = 0.460
	MEP area (mV ms)	5.12 ± 3.01	4.21 ± 1.66	4.52 ± 1.83	Friedman's test	χ^2^ = 4.167, *P* = 0.125
	*M*_max_ amplitude (mV)	19.4 ± 3.48	18.95 ± 3.12	16.91 ± 3.11	Friedman's test	χ^2^ = 2.000, *P* = 0.368
	*M*_max_ area (mV ms)	59.29 ± 13.88	60.23 ± 14.23	52.36 ± 10.99	Friedman's test	χ^2^ = 0.667, *P* = 0.717
**AP**	MEP amplitude (mV)	0.59 ± 0.28	0.6 ± 0.77	0.48 ± 0.26	Friedman's test	χ^2^ = 1.500, *P* = 0.472
	MEP area (mV ms)	2.32 ± 1.53	2.31 ± 2.14	2.75 ± 2.42	Friedman's test	χ^2^ = 0.167, *P* = 0.920
	*M*_max_ amplitude (mV)	4.68 ± 1.94	5.14 ± 2.19	4.77 ± 2.05	ANOVA	*F* (2, 18) = 0.279, *P* = 0.760
	*M*_max_ area (mV ms)	13.87 ± 5.71	13.16 ± 6.2	13.47 ± 6.42	Friedman's test	χ^2^ = 0.800, *p* = 0.670

*Note*: Baseline values for each parameter were compared across the three conditions (AIH, SHAM, AIC) using repeated‐measures one‐way ANOVA for normally distributed data (raw or log‐transformed) and Friedman's tests for data that were not normally distributed. A significance level of *P* < 0.05 was used. There was no difference in the baseline values for any parameter between the three days (AIH, SHAM and AIC).

### 3.2 Cardiorespiratory measures

Figure 2 shows the cardiorespiratory measures taken during AIH and AIC. During the hypoxic periods of the AIH protocol, the mean (SD) $F_{iO_{2}}$ was 9.3 (0.2)%, and during the hypercapnic periods of the AIC protocol, the mean (SD) $F_{\mathrm{iC}O_{2}}$ was 7.0 (0.5)%. AIC induced the greatest average increase in ventilation from ∼11 L/min to ∼15 L/min in an epoch. It was ∼25% greater than AIH (EMM [CI]: 2.1 [1.6 to 2.6] L/min, *P* < 0.001) and ∼34% greater than SHAM (3.6 [3.2 to 4.1] L/min, *P* < 0.001). AIH also increased ventilation more than SHAM by ∼16% (1.5 [1.1 to 1.9] L/min, *P* < 0.001). The increase in ventilation was driven by tidal volume, which was increased during the AIC by ∼44% (0.310 [0.272 to 0.348] L, *P* < 0.001) and AIH by ∼20% (0.145 [0.112 to 0.179] L, *P* < 0.001) compared to SHAM. Tidal volume was also higher during AIC than during AIH (0.165 [0.124 to 0.206] L, *P* < 0.001). Respiratory rate dropped during both AIC by ∼5% (−1 [−1.7 to −0.4] breaths/min, *P* < 0.001) and AIH by ∼2% (−0.7 [−1.3 to −0.1] breaths/min, *P* = 0.026) compared to SHAM with no difference between AIC and AIH (0.4 [−0.2 to 0.9] breaths/min, *P* = 0.184). Heart rate increased by ∼5% during both the AIC (3.7 [2.6 to 4.8] beats/min, *P* < 0.001) and AIH (4 [2.8 to 5.2] beats/min, *P* < 0.001) compared to SHAM. However, there was no difference in heart rate between AIH and AIC (0.3 [−0.6 to 1.3] beats/min, *P* = 0.503).

During AIC, the inspired CO_2_ ($F_{\mathrm{iC}O_{2}}$) of 7.04 (0.5)% produced an increase of ∼43% in $ET_{CO_{2}}$ compared to SHAM (2.1 [1.9 to 2.2]%, *P* < 0.001) with the lowest $S_{pO_{2}}$ at 98 (1)%. During AIH, the inspired O_2_ ($F_{iO_{2}}$) of 9.3 (0.2)% induced an average drop in $S_{pO_{2}}$ to ∼83 (4)% in each epoch, but there was no change in $ET_{CO_{2}}$ during AIH compared to SHAM (0.0 [−0.1 to 0.1]%, *P* = 0.733). There was no difference in ventilation between the 3 min before the breathing protocol and the 3 min after the breathing protocol on the AIH (*P* = 0.117) or AIC (*P* = 0.171) days.

### 3.3 Voluntary activation

Figure 3a shows voluntary activation pre‐ and post‐intervention across the three days (AIH, SHAM, AIC). For voluntary activation, there was an effect of condition (*F* (2, 608) = 19.76, *P* < 0.001). AIC was 5.9 [3.4 to 8.4]% higher than SHAM (*P* < 0.001) and 5.5 [3.1 to 7.8]% higher than AIH (*P* < 0.001). However, there was no difference between AIH and SHAM (0.5 [−1.6 to 2.5], *P* = 0.662). There was also no effect of time (*F* (19, 608) = 0.962, *P* = 0.506) nor an interaction of condition and time (*F* (38, 608) = 1.031, *P* = 0.421). Outlier removal did not alter the main effects.

### 3.4 MVC force

For MVC force, there was also an effect of condition (*F* (2, 608) = 6.77, *P* = 0.001; Figure 3b) with MVC force 7.7 [2.5 to 12.7]% higher on the AIC day than the SHAM day (*P* = 0.001). There were no significant differences between AIH and SHAM (3.6 [−0.8 to 7.9]%, *P* = 0.118) or AIC and AIH (4.1 [−0.8 to 9.0]%, *P* = 0.118). MVC force reduced with time on all three days (*F* (19, 608) = 3.699, *P* < 0.001) with significant reductions from baseline at all time points from 48 min post‐intervention (11.3 [2.1 to 20.4]%, *P* = 0.001) to 80 min post‐intervention (12.2 [5.2 to 19.3]%, *P* < 0.001). However, there was no interaction between the condition and time (*F* (38, 608) = 1.106, *P* = 0.308). Outlier removal did not alter the main effects.

### 3.5 Resting twitch

The resting twitch decreased from baseline after the intervention protocols on all three days (*F* (19, 608) = 4.643, *P* < 0.001; not illustrated) with significant decreases at 12 min after the interventions (5.6 [0.9 to 10.4]%, *P* = 0.003) and all points from 44 min (8.5 [0.3 to 16.6]%, *P* = 0.029) to 80 min post intervention (10.6 [3.6 to 17.6]%, *P* < 0.001). The maximal decrease of 11 [3.4 to 18.6]% was at 72 min post‐intervention (*P* < 0.001). There was no effect of condition (*F* (2, 608) = 1.248, *P* = 0.288) nor an interaction of condition and time (*F* (38, 608) = 0.706, *P* = 0.908).

### 3.6 EMG_rms_ in AP MVCs

There was an effect of condition on the EMG_rms_ recorded from AP during MVCs (*F* (2, 566) = 10.564, *P* < 0.001; Figure 3c). The EMG_rms_ in AP on the AIH day was higher by 14.3 [6 to 22.6]% than the SHAM (*P* < 0.001) and by 13.5 [5.3 to 21.6]% than the AIC day (*P* < 0.001). There was no difference between the SHAM and AIC day (0.8 [−6.3 to 8]%, *P* = 0.818). There was no effect of time (*F* (19, 566) = 0.43, *P* = 0.984) nor an interaction of condition and time (*F* (38, 566) = 0.343, *P* = 1).

### 3.7 *M*_max_ in AP and FDI

For the *M*_max_ amplitude and area for AP there were no effects of condition (amplitude: *F* (2, 174) = 2.136, *P* = 0.121; area: *F* (2, 174) = 1.392, *P* = 0.251), time (amplitude: *F* (5, 174) = 2.311, *P* = 0.046; area: *F* (5, 174) = 0.926, *P* = 0.466) or their interaction (amplitude: *F* (10, 174) = 0.315, *P* = 0.976; area: *F* (10, 174) = 0.1, *P* = 0.999).

For the *M*_max_ amplitude and area for FDI also there were no effects of condition (amplitude: *F* (2, 198) = 1.323, *P* = 0.269; area: *F* (2, 198) = 0.815, *P* = 0.444), time (*F* (5, 198) = 0.440, *P* = 0.820; area: *F* (5, 198) = 0.192, *P* = 0.965) or their interaction (amplitude: *F* (10, 198) = 0.374, *P* = 0.957; area: *F* (10, 198) = 0.303, *P* = 0.980).

### 3.8 MEPs evoked by TMS in FDI and AP

Non‐normalised MEPs in FDI grew over time. MEP amplitude showed an effect of time (*F* (5, 197) = 2.569, *P* = 0.028) across 3 days (AIH, SHAM, AIC) with a significant increase from ∼8 min post‐intervention to 80 min post‐intervention (0.453 [0.058 to 0.847] mV, *P* = 0.012). There was no effect of condition (*F* (2, 197) = 1.037, *P* = 0.356) as shown in Figure 4a, nor an interaction of condition and time (*F* (10, 197) = 0.798, *P* = 0.631). MEP area showed similar effects of time (*F* (5, 197) = 2.744, *P* = 0.02) with similar *post hoc* comparisons (80 min greater than the ∼8 min post‐intervention measure: 0.002 [0 to 0.003] mV ms, *P* = 0.008). Results were also similar when the amplitude and area were normalised to the corresponding *M*_max_ in FDI.

Non‐normalised MEPs in AP were unchanged throughout the experimental sessions. There was no effect of condition (*F* (2, 193) = 0.365, *P* = 0.694; Figure 4b), time (*F* (5, 193) = 0.566, *P* = 0.726) nor an interaction of condition and time (*F*(10, 193) = 0.594, *P* = 0.818) on the amplitude of the MEP in AP on the three days (AIH, SHAM, AIC). Similar results were also observed for the area of the MEP, and when the amplitude and area were normalised to the corresponding *M*_max_ in AP.

## 4 DISCUSSION

In this study, we used motor performance and neurophysiological measures to quantify hand motor output after AIH and AIC. AIC resulted in positive effects relative to the SHAM protocol on MVCs of adductor pollicis. However, the effects were small and not consistent across all parameters assessed. Our results show that voluntary activation after AIC was 5–6% higher than after both SHAM and AIH. In addition, maximal voluntary force was ∼8% higher after AIC than SHAM, but EMG during the MVCs was ∼14% higher after AIH than SHAM. Overall, there were 11–12% decreases in MVC force and resting twitch amplitude with time on all three days, suggesting that AIC preserved strength and AIH preserved EMG. The reductions in MVC and resting twitch suggest that the study protocol (MVC of adductor pollicis every 4 min) or the experimental set‐up may have interfered with muscle blood flow or induced a reduction in muscle force production capacity over time, despite previous studies using shorter rest times (1–2 min) between maximal contractions seeming to avoid fatigue (e.g., Allen et al., 1997, 1994). Thus, the improved voluntary activation after AIC tended to counteract the protocol‐related reductions in MVC force rather than improve force over baseline. By contrast, corticospinal excitability, tested with TMS‐evoked MEPs, was not affected by either AIH or AIC. Together, the results suggest that AIC may improve motor output to the hand in neurologically intact humans without SCI, but do not provide evidence for a specific neurophysiological mechanism.

### 4.1 Effectiveness of interventions

Intermittent periods of hypoxia and hypercapnia both showed expected physiological cardiorespiratory effects. During the AIH intervention, oxygen saturation dropped to ∼83% in each epoch and ventilation increased by ∼15%, which is similar to the three other studies that have reported ventilation during AIH with similar protocols (21–30%) (Finn et al., 2022; Mathew et al., 2024; Welch et al., 2022). Also consistent with these studies is that the increased ventilation was driven by increased tidal volume (by ∼20%) rather than respiratory rate (Finn et al., 2022; Mathew et al., 2024; Welch et al., 2022).

During AIC, increased inspired CO_2_ produced a mean increase in ventilation twice that produced by AIH, but that was still in the range of ventilatory increases achieved in response to AIH in previous studies (Finn et al., 2022). Also, similar to AIH, the AIC‐induced increase in ventilation was driven by an increase in tidal volume (by ∼44%). A small reduction in respiratory rate (by 1 breath/min) was observed during the hypercapnic periods of AIC, consistent with the reduction in respiratory rate observed after an initial increase in response to intermittent hyperoxic hypercapnia paradigms (Gozal et al., 1995).

### 4.2 Small improvements in hand motor output after single session of AIC

This study is the first to explore limb motor outcomes following intermittent exposure to inspired carbon dioxide (AIC) in neurologically intact humans. AIC increased voluntary activation by ∼6% relative to SHAM, confirming our hypothesis that AIC would improve motor output. Since maximal voluntary force of thumb adduction reduced with time after all three intervention protocols, the higher voluntary activation after AIC was supported by a preservation of maximal voluntary force of thumb adduction (∼8% higher after AIC compared to SHAM). However, there was no increase in maximal EMG. A poor relationship between surface EMG and force output is common at high muscle forces, where phase cancellation between motor unit action potentials and motor unit synchronisation affect EMG amplitude (Keenan et al., 2005). Indeed, the post‐AIC results contrast with the post‐AIH results, where EMG was increased but not force. While no other studies have examined the effects of AIC on limb muscles, the effect of intermittent hypercapnia on ventilation has been examined previously. In humans, there was no respiratory facilitation after 40 min of cycles of 40‐s hypercapnia (+5 mmHg above baseline end‐tidal $P_{CO_{2}}$)/20 s normocapnia in wakefulness (Shing et al., 2023), whereas longer periods of intermittent hypercapnia in animal models (at 10–15% CO_2_) generally have induced respiratory depression (Bach & Mitchell, 1998; Baker et al., 2001; Stipica Safic et al., 2018; Valic et al., 2016). In contrast, one study reported that daily AIC training with 7% hypercapnia over 2 weeks facilitated diaphragm EMG in rats (Randelman, 2021). Thus, AIC may act differently from AIH on respiratory muscles but may depend on the dose to engage different mechanisms in its actions on limb muscles. In the current study, ventilation in the first 3 min after the AIC or AIH intervention was not different from the SHAM intervention. However, this may not be sufficient time to demonstrate the neuroplastic changes in ventilation described in animals. Also, similar to AIH, AIC did not induce any facilitation or inhibition of MEP size, so the results provide little evidence about mechanisms other than that the increase in maximal force after AIC resulted from increased neural drive to the muscle, as shown by the increased voluntary activation.

### 4.3 Single session of AIH did not improve hand motor function

This study measured motor performance outcomes in response to a single session of AIH in adult humans without SCI, and demonstrated no improvements in motor output after AIH (compared to in people with SCI, Afsharipour et al., 2023; Pearcey et al., 2024; Sandhu et al., 2019, 2021; Trumbower et al., 2017, 2012, 2022). This absence of motor improvements is contrary to the ∼82% increase in ankle plantar flexion torque, ∼83% increase in gastrocnemius EMG activity (Trumbower et al., 2012), ∼21% increase in ankle plantar flexion torque after AIH and 41% increase after AIH + prednisolone (Sandhu et al., 2019) in people with SCI. A recent study in a neurologically intact population demonstrated preservation of motoneurone output compared to baseline (using the central activation ratio – ratio of superimposed twitch force and MVC, which does not take into account the resting twitch) without a corresponding change in strength during a fatigue protocol performed after 4 days of daily AIH (Bogard et al., 2024). However, this study is difficult to interpret as the effects after AIH were not compared with a sham protocol.

With voluntary activation of ∼92% at baseline before the AIH intervention, which is typical for neurologically intact people (Herbert & Gandevia, 1996), improvements from increased neural drive to the muscle are possible but somewhat limited by the voluntary activation ceiling of 100%. Moreover, the measurement of voluntary activation via superimposed twitches is relatively insensitive to changes in excitation at high contraction intensities (Herbert & Gandevia, 1999). Thus, small increases in neural drive may not translate to measurable increases in voluntary activation after AIH in a population with high voluntary activation at baseline. Nonetheless, improvements were seen after AIC with similar baseline voluntary activation.

Neither MEPs from FDI nor MEPs from AP were different after AIH compared to SHAM, although MEPs from FDI increased in size with time after all three interventions, while MEPs from AP did not. The lack of difference in MEP size between AIH and SHAM is consistent with current studies in neurologically intact participants of which four studies show that a single session of AIH has no effect on MEP size in hand, leg and diaphragm muscles (Mathew et al., 2024; Radia et al., 2022; Welch et al., 2022, 2021), while one other study shows an isolated increase in one parameter at one time point (Finn et al., 2022). These studies contrast with the facilitation of MEPs after a single session of AIH in two studies in the hand – one in neurologically intact participants without SCI (Christiansen et al., 2018) and one in people with SCI (Christiansen et al., 2021). In addition, after five daily sessions of AIH in neurologically intact participants, tibialis anterior MEPs increase compared to sham (Bogard et al. 2025). MEP size depends on the excitability of neurons in the cortex and the spinal cord, as well as the efficacy of the synapses connecting them (e.g., Rothwell, 1997; Rothwell et al., 1991; Spampinato et al., 2023). The current working hypothesis is that AIH‐induced motor facilitation occurs post‐synaptically at the motoneurones (Christiansen et al., 2018; Dale‐Nagle et al., 2010a, 2010b). Thus, an absence of change of MEPs after a single session of AIH in the current study and other studies in neurologically intact people fails to support this hypothesis. However, concurrent decreases in the excitability of cortical or spinal neurons could counter increases in synaptic efficacy and cannot be excluded. Moreover, multiple exposures to AIH might be needed to produce observable changes in MEP size.

There were no increases in the *M*_max_ on any of the intervention days, in contrast to our two previous studies (Finn et al., 2022; Mathew et al., 2024). This may be due to the current protocol, including the performance of regular MVCs, whereas in the other two studies, participants remained at rest. *M*_max_ has been shown previously to change over time when participants are at rest for prolonged periods (Cupido et al., 1996; Lefebvre et al., 2004).

There are other factors that are reported to influence the strength of motor facilitation after a single session of AIH. For example, female sex (e.g., Nair et al., 2023; Tester et al., 2013; cf. Vermeulen et al., 2020) and obstructive sleep apnoea (Vivodtzev et al., 2020) have been suggested to reduce AIH‐induced motor facilitation, while genetic factors (APOE4 with AIHH; Nair et al., 2023) and drugs like ibuprofen (Lynch et al., 2017), prednisolone (Sandhu et al., 2019) and caffeine (Trumbower et al., 2022) are speculated to augment AIH‐induced facilitation. It has also been hypothesised that there is reduced motor facilitation after AIH in neurologically intact individuals (Nair et al., 2023), but this is contrary to other studies that demonstrate motor facilitation in this population (e.g., Christiansen et al., 2018). In our study, 8 of the 13 participants were females, who have been suggested in some studies to show poor responses to AIH. We did not control or restrict caffeine or medications, but excluded people on serotonergic drugs and advised all participants to keep the same routine of food, exercise and caffeine on all experimental days. Though studies in rats suggest an influence of the time of day when the experiment is conducted (Fields & Mitchell, 2017; Hoffman et al., 2010; Perim et al., 2018), the evidence is mixed and suggests that there are responses to AIH during both the rest and active phase in rats, but peak effects are during the rest phase (Marciante et al., 2023).

### 4.4 Acute intermittent hypoxia, hypercapnia and hypercapnic hypoxia

Mechanisms for AIH have been well‐described through animal and cellular models (for reviews, see Mitchell & Johnson, 2003; Mitchell et al., 2001). Changes in arterial blood oxygen are sensed by the carotid bodies, which in turn activate the medullary raphe nuclei (for review see Lindsey et al., 2018). The serotonergic neurons of the raphe nuclei project to spinal motoneurones, and intermittent release of serotonin sets in play neuroplastic changes that modulate the responses of the motoneurones to excitatory input along with competing adenosinergic pathways (e.g., Hoffman & Mitchell, 2011; MacFarlane et al., 2008; Perim et al., 2019). These neuroplastic changes occur at the excitatory glutamatergic synapses on the phrenic motoneurones (Dale‐Nagle et al., 2010a, 2010b) and were seemingly supported by the initial findings of Christiansen et al. (2021, 2018) for a hand muscle in people with and without SCI. In humans, these neuroplastic changes should increase the size of evoked responses such as MEPs that act via these synapses (e.g., Rothwell, 1997; Rothwell et al., 1991), but in the current study, MEPs were not increased.

Hypercapnia, on the other hand, directly stimulates neurons in the raphe nuclei, increasing their firing (for review, see Kanamaru & Homma, 2007; Teran & Richerson, 2020), as well as acting indirectly via peripheral chemoreceptors (Morris et al., 1996). Thus, intermittent hypercapnia might be expected to induce serotonin‐dependent neuroplasticity at the motoneurones via similar molecular signalling mechanisms to AIH. However, these pathways with AIC have not been studied, and as noted above, intermittent hypercapnia depresses ventilation long‐term in contrast to the long‐term facilitation of ventilation by AIH (Bach & Mitchell, 1998; Baker et al., 2001; Stipica Safic et al., 2018; Valic et al., 2016).

As the current study found no facilitation of MEPs after either AIH or AIC, it does not provide evidence to support the proposed mechanism of synaptic plasticity in humans, but does not specifically exclude this mechanism. Other speculative explanations for increased voluntary motor output with no change in evoked potentials include increases in persistent inward currents in the motoneurones and/or facilitatory plasticity in the descending serotonergic system. Persistent inward currents (PIC) in spinal motoneurones are highly serotonin sensitive and are critical for the production of strong voluntary contractions (e.g., Heckman et al., 2005) but are not detectable with the use of evoked potentials as the synchronised excitatory input apparently activates motoneurones too quickly at rest (Thorstensen et al., 2022). A different possibility is that there are ‘upstream’ (e.g., pre‐primary motor cortex) contributions that are responsive to AIH and/or AIC such that motor cortical output is increased during voluntary contraction, but excitability is not altered with the muscles tested at rest (e.g., Kress & Mennerick, 2009).

Hypercapnia also increases the sensitivity of the carotid bodies to hypoxia (for review, see Kumar & Prabhakar, 2012). The mix of hypoxia with hypercapnia has been suggested to induce more consistent respiratory facilitation, with one study demonstrating increased corticospinal excitability of the diaphragm after AIHH but not after AIH (Welch et al., 2022). By contrast, other studies suggest the absence of respiratory long‐term facilitation after intermittent hypercapnic hypoxia in both awake (Diep et al., 2007) and asleep individuals (Deacon et al., 2017). It is also proposed that to induce respiratory long‐term facilitation with AIH, it must be delivered under continuous hypercapnic conditions (Mateika et al., 2024). However, continuous hypercapnia has not been required to elicit AIH‐induced increases in upper limb (hand: Sandhu et al., 2021; arm: Afsharipour et al., 2023) and lower limb muscle function after SCI (Hayes et al., 2014; Lynch et al., 2017; Sandhu et al., 2019; Tan et al., 2021; Trumbower et al., 2012).

Overall, the net effect of intermittent hypoxia, intermittent hypercapnia or hypercapnic hypoxia on human motor function (respiratory and limb muscles) in humans is still unclear. Limb motor outcomes after AIH have been inconsistent within and between different studies, showing large improvements, small improvements, and even no improvement of limb muscle output (including the current study) (Afsharipour et al., 2023; Pearcey et al., 2024; Sandhu et al., 2019, 2021; Trumbower et al., 2017, 2012, 2022). In addition, most of the evidence suggests a lack of facilitation of limb and respiratory corticospinal excitability evoked through magnetic stimulation (Finn et al., 2022; Mathew et al., 2024; Radia et al., 2022; Welch et al., 2022, 2021; cf. Christiansen et al., 2021, 2018). From the current study, AIC increased voluntary activation and MVC force compared to SHAM but, like AIH, showed no evident MEP increase. AIHH, on the other hand, can induce increased facilitation of the motor pathway detected by MEPs from the diaphragm and initial respiratory drive by measuring mouth pressure 0.1 s after the onset of an occluded inspiration in humans (Welch et al., 2022) but does not lead to functionally meaningful respiratory facilitation overall (Deacon et al., 2017; Diep et al., 2007). It is possible that different levels of serotonergic secretion at different levels of the central nervous system could activate competing serotonergic mechanisms for neuroplasticity, resulting in inconsistent or absent motor facilitation (e.g., cross‐talk inhibition) (Perim et al., 2017, 2018). The dosage (exposure duration and level of $F_{iO_{2}}$ and $F_{\mathrm{iC}O_{2}}$) for an individual may need to be optimised to evoke maximal motor facilitation.

### 4.5 Limitations

In our study, participants were neurologically intact, which may affect the likelihood of observing larger improvements in motor output. Also, we delivered only a single session of AIH and AIC, which may limit the magnitude of hypothesised responses. Our sample size was calculated based on a relatively large effect size of a single session of AIH; however, the effect size on motor output is potentially much smaller after a single session. In addition, we had predominantly female participants (8 of 12). There is some evidence that females show smaller motor improvements after AIH (Nair et al., 2023), although increases in ventilation seem to be similar in males and females following AIHH (Vermeulen et al., 2020). In this study, we used a fixed dose AIH and AIC; however, there are limited data on doses that consistently evoke maximal motor facilitation in humans.

### 4.6 Conclusion

This study demonstrated functional motor facilitation after AIC but not AIH on a background of declining muscle force in a group of participants without SCI. This facilitation was small but was in a healthy motor‐intact group that had less potential for improvement. The effects of AIH, AIC and AIHH paradigms on functional motor facilitation need to be explored further in humans with greater potential for improvement, for example, people with incomplete spinal cord injuries or stroke. MEPs provided no evidence of synaptic plasticity of the motor pathway after either AIH or AIC protocols, and this is consistent with a growing number of studies. Our findings question the mechanisms currently hypothesised for AIH‐induced improvements in function in humans and raise questions around dosage and the need for tailored interventions.

## AUTHOR CONTRIBUTIONS

Anandit J. Mathew, Harrison T. Finn, Simon C. Gandevia, Janet L. Taylor, and Jane E. Butler conceived and designed research; Anandit J. Mathew, Harrison T. Finn, and Chiettha Prajnadewie performed experiments; Anandit J. Mathew and Harrison T. Finn analysed data; Anandit J. Mathew, Harrison T. Finn, Simon C. Gandevia, Janet L. Taylor, and Jane E. Butler interpreted results of experiments; Anandit J. Mathew prepared figures; Anandit J. Mathew drafted manuscript; Anandit J. Mathew, Harrison T. Finn, Chiettha Prajnadewie, Simon C. Gandevia, Janet L. Taylor, and Jane E. Butler edited and revised manuscript. All authors have read and approved the final version of this manuscript and agree to be accountable for all aspects of the work in ensuring that questions related to the accuracy or integrity of any part of the work are appropriately investigated and resolved. All persons designated as authors qualify for authorship, and all those who qualify for authorship are listed.

## CONFLICT OF INTEREST

The authors declare they have no conflicts of interest.

## Data Availability

The data are available on request from the corresponding author.
